# The Territorial Regimental Surgeon and First-Aid Instruction

**Published:** 1909-04-24

**Authors:** 


### The Royal Army Medical Corps Section.

### THE TERRITORIAL REGIMENTAL SURGEON AND FIRST-AID INSTRUCTION.

Of the three lines of medical assistance given to the wounded in war, regimental aid is the first, and it falls to the regimental surgeon to train properly the men allocated for this work. Formerly two men were furnished by each company for it, but under the new Territorial Regulations it is laid down that one corporal and 16 bandsmen are to be detailed for this duty. To enable him to carry out the tuition of these men satisfactorily, as they will require some instruction in the leading facts of anatomy and physiology, the regimental surgeon will need diagrams and a skeleton, and for the loan of these he should make application to his Divisional School of Instruction. For the temporary use of a fracture box a requisition should be made to the A.M.O. of the division, as also for any bandages or other instructional stores required. The arrangement for the course and the hours of attendance should be made through the commanding officer of the regiment, so that the meetings of the class will not clash with the other engagements of the bandsmen.

As regards the scope of the instruction to be given, the regimental surgeon should bear in mind that the men of the class are being trained for a particular service and not for the general emergencies of every-day life. That particular service consists in being able to render prompt and proper first aid to the wounded, be they comrades or enemies. The basis of all such instruction should be that first aid has certain definite and fundamental objects, and that nowhere are these more clearly marked out than on the battle-field. Briefly they are the following: (1) The saving of life as by the arrest of bleeding; (2) protection against further aggravation of injury, as in the case of wounds and fractures; (3) relief of pain; (4) prevention of any unnecessary suffering, as from rough handling and moving. If the regimental surgeon will make the above four objects the groundwork of his teaching he will furnish instruction on the most important and necessary details of which regimental stretcher-bearers should have knowledge. Any regiment that has a body of men attached to it that are well-grounded in the arrest of hæmorrhage, are adepts in applying the first field dressing, and the use of fracture apparatus, and are skilful in the loading, carrying, and unloading of stretchers is possessed of efficient regimental aid. In the management of his class the regimental surgeon should remember that the non-medical mind is apt to be disheartened when it pictures, according to its own ideas, what first aid implies, but if it can be got to realise that the field of work involved in regimental aid is limited and that attention to certain clearly-marked lines of procedure can be the means of doing an incalculable amount of good this furnishes a stimulus and encouragement that are invaluable. Accordingly he should impress on his stretcher bearers how important their services are in the ultimate issue of the case. Further, he should keep constantly before them that what is needed to make them proficient is steady application to their duties, so that they may gain the needed manipulative skill, rapidity of work being very desirable. In regimental aid time is everything in dealing with the wounded in a battle. If manual dexterity is wanting, much time is consequently spent on individual cases, so that the stretcher-bearers will be unable, from physical exhaustion alone, to meet all demands.

In dealing with the temporary arrest of hæmorrhage, the regimental surgeon should draw attention to the need for great care in this matter. There is a prevalent idea that all that is needed is the application of a tourniquet, but it should be pointed out that this latter is a mechanical contrivance, and that if precautions are not taken serious injury may be done to underlying structures if it is injudiciously applied. In the same way the stretcher-bearers should be got to realise how much the course of a wound depends in many cases upon its first treatment, and that consequently there is need for a proper dressing and for great care in its application. On these grounds the value of the first field dressing supplied in his kit to every British soldier on active service, should be emphasised and the importance of knowing how to use it and apply it. Again, when dealing with gun-shot fractures the regimental surgeon should not lose sight of the fact that occasions arise when regular splints are not available, and that it is necessary sometimes to improvise extempore ones from materials available on the battlefield. Examples of the articles that might be so utilised should be given, and specimens of them should be shown as well as the recognised rifle splint for fractured thigh.

Important, however, as are the arrest of bleeding and the immobilisation of fractures in first aid, a matter of equal moment is the prevention of unnecessary suffering to the wounded by having them gently and carefully handled. Nothing is worse for an injured person than rough and needless movement. Hence the imperative necessity for the very thorough training of the stretcher-bearers in the loading, carrying, and unloading of stretchers with wounded on them. The new stretcher drill with six men in a squad cannot be followed in the case of regimental stretcher-bearers. Nor is it needed, because, as a rule, they will not be called upon to carry the wounded any distance. All that will be required of them is to place them in a position of shelter where they can be left until picked up by the bearer division of the field ambulance. In view of this, their stretcher drill should be that given in the manual with reduced numbers, and they should practise the exercises as laid down for four, three, or even two bearers. This will meet their case, and if they are also given a knowledge of the formation of hand-seats they will be sufficiently trained to meet any variety of circumstances that may arise.

The regimental surgeon, however, should not be content with the proficiency of the stretcher-bearers only in the drill-hall or on the parade ground. He should take every opportunity of exercising them under such conditions as would actually occur in warfare or in peace during training in field work.

